# Tnni3k regulates cardiomyopathy and cardiac conduction disease through Nfatc1 signaling

**DOI:** 10.1016/j.gendis.2024.101464

**Published:** 2024-11-13

**Authors:** Shi Ouyang, Yujuan Niu, Le Liu, Qiaorong Yi, Wuming Qin, Hui Cao, Tao Liao, Rong Xiang, Yonghe Ding, Yun Deng

**Affiliations:** a State Key Laboratory of Developmental Biology of Freshwater Fish, Hunan Normal University, Changsha, Hunan 410081, China; b Laboratory of Zebrafish Genetics, College of Life Sciences, Hunan Normal University, Changsha, Hunan 410081, China; c The Affiliated Hospital of Qingdao University, Qingdao University, Qingdao, Shandong 266071, China; d The Biomedical Sciences Institute of Qingdao University (Qingdao Branch of SJTU Bio-X Institute), Qingdao University, Qingdao, Shandong 266071, China; e NHC Key Laboratory of Birth Defect for Research and Prevention, Hunan Provincial Maternal and Child Health Care Hospital, Changsha, Hunan 410028, China; f Development of Cell Biology, The School of Life Sciences, Central South University, Changsha, Hunan 410083, China

*TNNI3K* (troponin-I interacting kinase) encodes a duo tyrosine and serine/threonine kinase implicated in cardiomyopathy, arrhythmias, and cardiac conduction disease (CCD).^1^ However, no direct downstream phosphorylation targets of TNNI3K have been identified yet.^2^ Here, we employed the CRISPR/Cas9 gene-editing technique to generate a splicing mutation in the 4th exon of zebrafish *tnni3k* ortholog gene that mimics a *TNNI3K* splicing variant identified from a patient family with cardiomyopathy and CCD. This *tnni3k* heterozygous mutant (*tnni3k*^*e4/+*^) recapitulated several key features of cardiomyopathy and CCD, thus representing a novel model of human TNNI3K mutation-based cardiac diseases. Next, we utilized this heterozygous *tnni3k*^*e4/+*^ mutant to perform proteomics and phosphoproteomic analysis. As a result, we identified Mypt1/Mlc2/Yap1/Nfatc1 axis as the downstream phosphorylation targets of Tnni3k. Lastly, we found that treatment of cyclosporine A, an inhibitor of Nfatc1 translocation from cytoplasm to the nucleus, exhibited a partial cardioprotective effect on the *tnni3k*^*e4/+*^ heterozygous mutant. Together, we generated a unique zebrafish animal model of TNNI3K-based cardiac diseases and identified the Mypt1/Mlc2/Yap1/Nfatc1 axis as its downstream phosphorylation targets that could be potentially leveraged to develop new therapeutic strategies.

The zebrafish *tnni3k* gene exhibits a cardiac-specific expression pattern and encodes a protein that shares approximately 85 % amino acid identity with human TNNI3K (Fig. 1A; Fig. S1), supporting TNNI3K as an evolutionally highly conserved protein across species. A previous study reported that a novel splice site mutation in the 4th exon of the human *TNNI3K* gene (c.333 + 2T > C) caused dilated cardiomyopathy and CCD.^3^ To mimic the cardiomyopathy and CCD caused by this human *TNNI3K* variant, we designed a single guide RNA targeting the splicing donor site of the 4th exon in the zebrafish *tnni3k* gene and generated a splicing mutant in the predicted splice donor site via CRISPR/Cas9 technology (Fig. 1B; Fig. S2A–C). This splicing mutation resulted in aberrant *tnni3k* splicing between exon 4 and exon 5 and led to a premature stop of Tnni3k protein translation. As a result, the mRNA level of *tnni3k* was significantly reduced in both the *tnni3k* heterozygous (*tnni3k*^*e4/+*^) and homozygous (*tnni3k*^*e4/e4*^*)* mutants compared with that in the wild-type sibling, likely due to a nonsense-mediated mRNA decay mechanism (Fig. 1C). Next, we carried out detailed phenotypic analysis on these *tnni3k* splicing mutants. First, we found T wave elevation in the *tnni3k* homozygous but not heterozygous mutant (Fig. 1D). Second, we detected significant cardiac function decline and heart rate reduction in both the *tnni3k*^*e4/+*^ and *tnni3k*^*e4/e4*^ mutants (Fig. 1E, F). Third, we found obvious increased myocardium muscle density, sarcomere degeneration, mitochondrial swelling, and/or Z-disc distortion phenotypes, most notably in the *tnni3k*^*e4/e4*^ *homozygous* mutant hearts (Fig. 1G, H). Collectively, we generated a splice site mutation in the zebrafish *tnni3k* gene that led to cardiac dysfunction and conduction disorder, recapitulating several key features of human TNNI3K mutation-caused cardiomyopathy and CCD.

**Figure 1:**
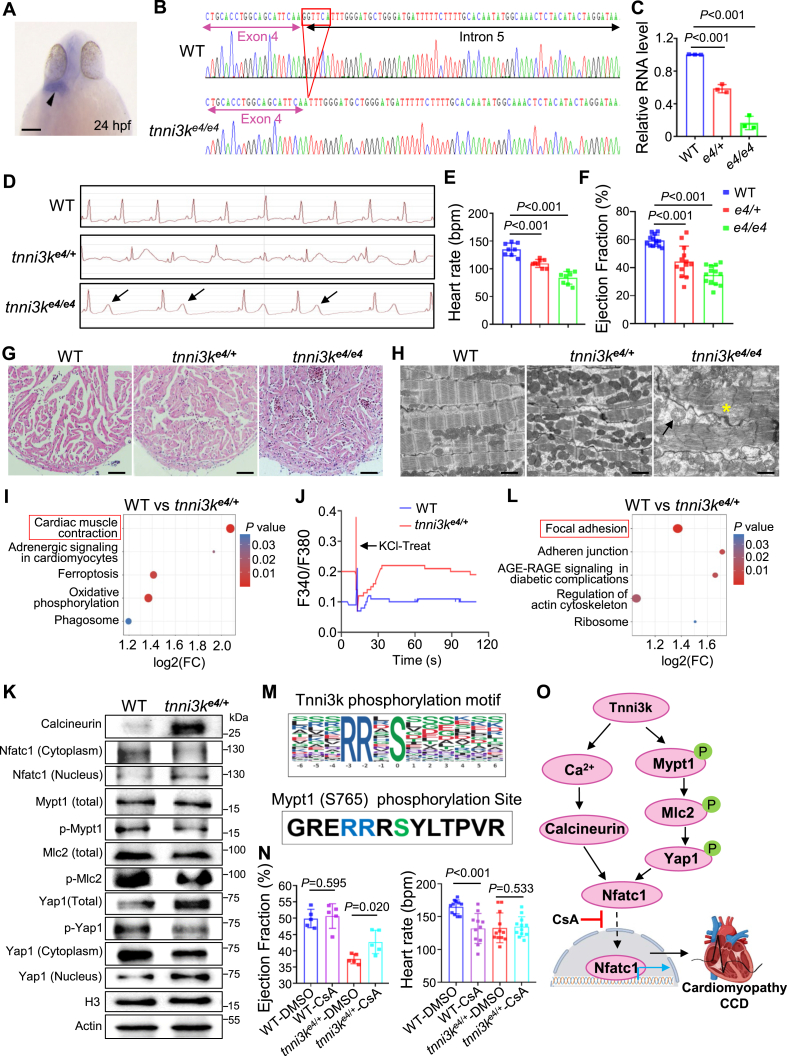
Tnni3k regulates cardiomyopathy and cardiac conduction disease through Nfatc1 signaling. **(A)** Whole-mount *in situ* hybridization (WISH) assay for detecting endogenous expression patterns of *the tnni3k* gene at 24 h post-fertilization (hpf). The black arrows point to the WISH signal in the heart. Scale bar, 100 μm. **(B)** The chromographs illustrating the sequences of the wild-type (WT) *tnni3k* gene and the mutant alleles with a 6-base pair (bp) nucleotide deletion (red box) in the splicing site adjacent between the 4th exon and 5th intron at the genomic levels. **(C)** The *tnni3k* transcript levels between WT and mutant alleles detected by quantitative real-time PCR. *n* = 3, one-way Analysis of Variance (ANOVA). **(D, E)** Representative electrocardiogram (ECG) recordings (D) and heart rate quantification analysis (E) in the *tnni3k* mutants and WT sibling controls at 6 months. The arrows indicate T-wave elevation. *n* = 8, one-way ANOVA. bpm, beats per minute. **(F)** Ejection fraction (EF) (in %) measured by echocardiography in the indicated *tnni3k* mutant lines and WT sibling controls at 6 months. *n* = 8–13, one-way ANOVA. **(G)** Representative hematoxylin & eosin (H&E) staining of the apex area of the ventricle in the *tnni3k* mutants and WT sibling controls at 6 months. Scale bars, 100 μm. **(H)** The confirmative transmission electron microscopy (TEM) images showing mitochondrial swelling/empty (arrow) and myofibril degeneration phenotype (asterisk) in the *tnni3k*^*e4/e4*^ homozygous, but not obvious in the *tnni3k*^*e4/+*^ heterozygous mutant hearts at 6 months. Scale bars, 5 μm. **(I)** The top five enriched Kyoto Encyclopedia of Genes and Genomes (KEGG) pathways with significant fold changes in expression levels and *P* values resulting from proteomic analysis between the *tnni3k*^*e4/+*^ mutants and WT sibling controls. The cardiac muscle contraction pathway was affected most significantly, and proteins in this pathway were subsequently experimentally tested. **(J)** Increased [Ca^2+^]_i_ was detected in the *tnni3k*^*e4/+*^ mutant cardiomyocytes compared with WT controls. **(K)** Representative western blotting images on the indicated protein expression levels in the *tnni3k*^*e4/+*^ mutant hearts compared with the WT sibling controls at 6 months. **(L)** The top five enriched KEGG pathways with significant fold changes in expression levels and *P* values resulting from phosphoproteomic analysis between the *tnni3k*^*e4/+*^ mutants and WT sibling controls. The focal adhesion pathway was highlighted, and proteins in this pathway were focused on with subsequent experimental validation. **(M)** Putative *Tnni3k* phosphorylation substrate motif was predicted using MoMo (http://meme-suite.org/tools/momo). **(N)** Percent ejection fraction measured by echocardiography and heart rate measured by ECG in the *tnni3k*^*e4/+*^ mutant fish compared with the WT sibling controls at 6 months with or without cyclosporine A (CsA) treatment. *n* = 5–10, one-way ANOVA. **(O)** Proposed working model. Tnni3k functions by regulating calcineurin/Nfatc1-mediated calcium homeostasis and phosphorylating members of the Mypt1/Mlc2/Yap1/Nfatc1 axis to maintain cardiac contraction and heart rhythm. CCD, cardiac conduction disease.

To identify downstream protein targets of Tnni3k phosphorylation and gain insights into the pathways regulated by the Tnni3k protein underlying the molecular mechanisms of aberrant *tnni3k* splicing-induced cardiomyopathy and CCD, we performed both quantitative proteomic and phosphoproteomic analysis using protein lysate isolated from the *tnni3k*^*e4*/+^ heterozygous mutant hearts, as the mimicking *TNNI3K* splice site variation identified from the human patient is a heterozygous mutation.

First, through the proteomic analysis, we identified 212 differentially expressed proteins in the *tnni3k*^*e4*/+^ hearts compared with the wild-type controls based on a fold change >1.3 and a *P* value < 0.05 (Fig. S3B). Kyoto Encyclopedia of Genes and Genomes (KEGG) enrichment analysis showed that the most prominently affected pathway was cardiac muscle contraction (Fig. 1I). As studies have suggested that cardiac muscle contraction is regulated mainly by the intracellular free Ca^2+^ concentration ([Ca^2+^]_i_),^4^ we then focused on its downstream cascade reaction of the Ca^2+^/calcineurin/Nfatc1 signaling pathway for further experimental validation. We examined [Ca^2+^]_i_ by imaging the intracellular calcium concentration and detected an increase of [Ca^2+^]_i_ in the *tnni3k*^*e4/+*^ splicing mutant cardiomyocytes compared with the wild-type controls (Fig. 1J). We then evaluated the total protein levels of the calcineurin and Nfatc1 proteins, which are two positive downstream effectors of Ca^2+^. Our results showed that calcineurin and nuclear Nfatc1 were significantly up-regulated, while cytoplasmic Nfatc1 (cytoplasm) was down-regulated in the *tnni3k*^*e4/+*^ mutant hearts compared with the controls (Fig. 1K; Fig. S3E). Thus, these proteomic analysis results indicated that Tnni3k might function by regulating cardiac muscle contraction and/or Ca^2+^/calcineurin/Nfatc1 homeostasis to maintain normal cardiac function and rhythm.

Second, through the phosphoproteomic analysis, we found that protein phosphorylation levels were increased at 102 sites of 83 proteins and decreased at 518 sites of 310 proteins. KEGG enrichment of the differentially expressed phosphorylated proteins showed that focal adhesion was among the most significantly affected pathways in the *tnni3k*^*e4/+*^ mutant hearts (Fig. 1L). We further performed the phosphorylation motif analysis which predicted that the Tnni3k target proteins carried arginine-arginine-X-serine amino acid residues (Fig. 1M). Interestingly, among the top differentially expressed phosphorylated protein targets identified as being associated with focal adhesion, Mypt1 carried the same phosphorylation site (S765) as the predicted targeting motif of the Tnni3k protein. Thus, we performed a more detailed experimental validation analysis on the Mypt1 protein and its downstream effectors, such as Mlc2 and Yap. Indeed, we confirmed that phosphorylated Mypt1 and Yap1 proteins were down-regulated significantly in the *tnni3k*^*e4/+*^ hearts compared with the wild-type controls, and subsequently, nuclear Yap1 were significantly up-regulated, while cytoplasmic Yap1 (cytoplasm) was down-regulated in the *tnni3k*^*e4/+*^ hearts (Fig. 1L; Fig. S3E), consistent with the data from the proteomic and phosphoproteomic analyses. Taken together, this phosphoproteomic analysis identified the Mypt1/Ml2/Yap1 axis as the likely downstream phosphorylation target of the Tnni3k protein.

Next, considering that Mypt1/Yap1 is an upstream effector of Nfatc1, regulating the translocation of Nfatc1 into the nucleus,^5^ we tested the hypothesis that pharmacologic blockade of Nfatc1 into the nucleus in the *tnni3k* mutant would confer a cardioprotective effect on the *tnni3k* splicing mutation-based cardiomyopathy and CCD. To do so, we treated the *tnni3k*^*e4/+*^ mutant and wild-type sibling control fish with cyclosporine A, an inhibitor of Nfatc1 in the nucleus, which acts by restraining the activity of Ca^2+^-dependent calcineurin. The results showed that cyclosporine A treatment indeed led to a statistically decreased level of protein in the nucleus in both the *tnni3k*^*e4/+*^ mutant and wild-type control hearts (Fig. S4). More importantly, cyclosporine A treatment partially restored the cardiac function decline, but seemed to have no impact on the heart rate reduction in the *tnni3k*^*e4/+*^ mutant (Fig. 1N). Thus, these data indicated that pharmacologic inhibition of Nfatc1 translocation from the cytoplasm into the nucleus through cyclosporine A treatment could exert a partial therapeutic effect on *tnni3k* splicing mutation-based cardiomyopathy.

In conclusion, we generated a unique zebrafish animal model of TNNI3K splicing mutation-based cardiomyopathy and CCD and identified the focal adhesion pathway and Mypt1/Mlc2/Yap1/Nfatc1 axis as the downstream phosphorylation targets of the Tnni3k protein. We found that pharmacological inhibition of the translocation of Nfatc1 into the nucleus partially alleviated the cardiomyopathy phenotypes in the heterozygous *tnni3k* splicing mutant zebrafish model (Fig. 1O). More studies are needed to validate these findings in large mammalian animal models and to develop NFATC1 nuclear translocation-targeted therapeutic strategies for TNNI3K gene mutation-based cardiomyopathy and CCD.

## Author contributions

**Shi Ouyang:** Conceptualization, Data curation, Formal analysis, Investigation, Methodology, Software, Validation, Visualization, Writing – original draft, Writing – review & editing. **Yujuan Niu:** Data curation, Formal analysis, Investigation, Methodology, Validation, Visualization, Writing – review & editing. **Le Liu:** Data curation, Methodology. **Qiaorong Yi:** Data curation, Methodology. **Wuming Qin:** Data curation, Formal analysis, Methodology. **Hui Cao:** Data curation, Methodology. **Tao Liao:** Data curation, Methodology. **Rong Xiang:** Data curation, Formal analysis, Validation. **Yonghe Ding:** Conceptualization, Formal analysis, Funding acquisition, Investigation, Project administration, Resources, Supervision, Visualization, Writing – original draft, Writing – review & editing. **Yun Deng:** Conceptualization, Funding acquisition, Investigation, Project administration, Resources, Supervision, Writing – original draft, Writing – review & editing.

## Ethics declaration

The animal study protocols were approved by the Institutional Animal Care and Use Committees of Hunan Normal University (No. 2019003) and Qingdao University (No. QDU-AEC-2023201).

## Funding

This study was supported in part by the 10.13039/501100001809National Natural Science Foundation of China (No. 31970504, 31772548, 82371863, 82070394), the NHC Key Laboratory of Birth Defect for Research and Prevention (Hunan Provincial Maternal and Child Health Care Hospital) (No. KF2021003), and the Postgraduate Scientific Innovation Fund of Hunan Province, China (No. CX2018B302).

## Conflict of interests

The authors declared no conflict of interests.
