# Supplementary material for: Tnni3k regulates cardiomyopathy and cardiac conduction disease through Nfatc1 signaling
Source: Genes Dis. 2024 Nov 13;12(3):101464. doi: 10.1016/j.gendis.2024.101464 (PMC11804685; doi:10.1016/j.gendis.2024.101464)
Supplement: Multimedia component 1 [file mmc1.docx]

**Supplementary data**

**Materials and Methods**

**Animals**

Zebrafish (*Danio rerio*) (TU strain) were obtained from the China Zebrafish Resource Center (Wuhan, China) and maintained on a 14 h light/10 h dark cycle at 28.5 °C. All animal study procedures were performed in accordance with the Guide for the Care and Use of Laboratory Animals published by the US National Institutes of Health. The animal study protocols were approved by the Institutional Animal Care and Use Committee of Hunan Normal University (No. 2019003).

**Generation of the *tnni3k* mutant line**

The *tnni3k* mutant line was generated by co-injection of a single guide RNA (sgRNA) and Cas9 protein into one-cell stage embryos. The sgRNA targeted to the 4^th^ exon of the zebrafish *tnni3k* gene (GGCAGCAUUCAAGGUUCAUUUGG) was synthesized in vitro using a MAXIscript™ T7 Transcription Kit (Invitrogen) according to the manufacturer’s instructions and then purified using a RNeasy Mini Kit (Qiagen). The Cas9 protein was TrueCut™ Cas9 Protein v2 (Invitrogen). Forty nanograms of sgRNA and 0.5 ng of the Cas9 protein complex were assembled by incubation at 37 °C for 10 min and then injected into one-cell stage embryos. The injected embryos were collected at 48 hours post-fertilization (hpf) for examination of sgRNA knockout efficiency using polymerase chain reaction (PCR) with primers (F: AAGCTCACATCAGGACCCTC, R: GTCCAGCACTCACCTCAAGA) and Sanger sequencing. To identify germline-transmitted mutations, the injected founder embryos (F0) were raised to adulthood and then crossed with wild-type (WT) embryos to generate stable heterozygous embryos (F1). Genotyping was performed by PCR using genomic DNA isolated from the caudal fin followed by Sanger sequencing. The F1 mutants carrying the same indel were incrossed to generate homozygous mutants (F2).

**Whole-mount in situ hybridization (WISH)**

WT embryos of the TU strain at 24 hpf were subjected to WISH^[1]^. Digoxigenin-labeled anti-sense *tnni3k* RNA probes were synthesized from PCR products using T7 RNA polymerase from the DIG-RNA labeling mix (Roche) according to the manufacturer’s instructions. The PCR primer sequences used for RNA probe synthesis were as follows: *tnni3k*-WISH-F: AAGCTCACATCAGGACCCTC,

*tnni3k*-WISH-R: TCTGTTCGTCCTTTTCCCCA.

**Reverse Transcription and Quantitative Real-Time PCR**

Zebrafish were anesthetized with 0.02% tricaine for 10-15 minutes, and then heart tissues of adult zebrafish were dissected and subjected to total RNA extraction using TRIzol (Thermo Fisher Scientific). Approximately 1 µg of total RNA was used for reverse transcription and cDNA synthesis using a RevertAid First Strand cDNA Synthesis Kit (Thermo Fisher Scientific) according to the manufacturer's instructions. Reverse transcription PCR (RT‒PCR) was performed to assess splicing mutations and *tnni3k* RNA levels. The intron retention event in zebrafish *tnni3k* mutants was detected with the following primers: *tnni3k*-RT-F1: TCAAGCATGTTTTCAGCTCAG, *tnni3k*-RT-R1: CTGCTCGTGGCCAAAGTAAG. The relative *tnni3k* transcript level was examined with the primers *tnni3k*-RT-F2: TGAGAGGTTAGAGGAGGACTTGA and *tnni3k*-RT-R2: CCACATATGCAGCAGAGATGA. Quantitative real-time PCR was run using a CFX Connect Real-Time System (BIO-RAD, USA) in a total volume of 10 µL of reaction solution containing 1x SYBR Green Master Mix (Yeasen Biotechnology, 11184ES08, China), 0.2 µM gene-specific primer pairs and 1 µl cDNA template. Three-step PCR with a 60 °C annealing temperature was used for all primers. Gene expression levels were normalized using the expression levels of *β-actin* according to the 2^–ΔΔCt^ (cycle threshold) values. The primer sequences were as follows: *tnni3k*-F: GCTGCATATGTGGAGGGAATAA, *tnni3k*-R: CAGTATATCCATTGCGGGTGAG; *β-actin*-F: CGTGACATCAAGGAGAAG, *β-actin*-R: GAGTTGAAGGTGGTCTCAT.

**Hematoxylin-Eosin (H&E) Staining**

Zebrafish were anesthetized with 0.02% tricaine for 10-15 minutes, and then heart tissues of adult zebrafish were harvested and transferred to 10% potassium chloride solution at end-diastole followed by fixation in 4% paraformaldehyde. Fixed hearts were embedded in paraffin and cut transversely into 5 µm sections. Serial heart sections were stained with H&E to examine cardiac muscle morphology. Images were analyzed using a quantitative digital image analysis system (Image-Pro Plus 6.0).

**Transmission Electron Microscopy**

Zebrafish were anesthetized with 0.02% tricaine for 10-15 minutes, and then heart tissues of adult zebrafish were dissected, fixed in Trump’s fixative solution (4% paraformaldehyde and 1% glutaraldehyde in 0.1 M phosphate buffer [pH 7.2]) at room temperature for 1 h and then incubated overnight at 4 °C. The fixed samples were subsequently processed and imaged using a HITACHI HT7700 transmission electron microscope.

**Electrocardiography (ECG)**

Zebrafish ECG was performed according to reported methods^[2]^. Briefly, one week before the ECG, fish were subjected to microsurgery under a dissection microscope to remove the silvery epithelial layer of the hypodermis. One week after recovering from microsurgery, the fish were anesthetized with 0.02% tricaine for 6 minutes and then subjected to two minutes of ECG recording using an iWorx Systems according to the instructions (ZS-200, iWorx Systems, Inc).

**Echocardiography**

Cardiac functional phenotypes were measured and analyzed using a Vevo 3100 high-frequency imaging system equipped with a 50 MHz linear array transducer (Fujifilm VisualSonics) based on a previously reported protocol^[3]^. Briefly, fish were anesthetized with 0.02% tricaine for 6 minutes and placed ventral side up in a small sponge holder submerged in a plastic plate filled with aquarium water. The ultrasound transducer (MX700) was positioned on the ventral side of the zebrafish to provide a sagittal-plane image of the heart. B-mode images were acquired with an imaging field of view of 9.00 mm in the axial direction and 5.73 mm in the lateral direction, a frame rate of 123 Hz, medium persistence and a transmit focus at the center of the heart. Image quantification was performed using the VevoLAB workstation. Cardiac function indices, including ejection fraction (EF) and fractional shortening (FS), were calculated using the formulas EF=(EDV−ESV)/EDV and FS=(Ld−Ls)/Ld, respectively. For each index in individual fish, measurements were performed on 3-5 independent cardiac cycles to acquire average values.

**Cardiomyocyte Dissociation and Intracellular Calcium Imaging**

Adult ventricular cardiomyocytes were obtained using enzymatic dissociation according to previously reported methods with minor modifications^[4]^. Briefly, fish were anesthetized with 0.02% tricaine for 10-15 minutes, and then ventricles dissected from fish hearts were transferred to 250 µL of solution A (containing 13.5 mM NaCl, 5.4 mM KCl, 1 mM MgCl_2_, and 10 mM HEPES) and incubated for 8 minutes at 37 °C before being enzymatically digested using 250 µL of solution B (solution A supplemented with 4 mg/mL collagenase I and 0.56 mg/mL protease) for 6-8 minutes at 37 °C 3-5 times. The digested cells were then pelleted by centrifugation at 2000 rpm for 2 minutes. The supernatant was removed, and the resulting pellet was resuspended in 1 mL L-15 Leibovitz Media (HyClone, SH30525.01) supplemented with penicillin (100 U/mL)/streptomycin (100 µg/mL). The cells were then cultured in L-15 Leibovitz media supplemented with penicillin (100 U/mL)/streptomycin (100 µg/mL) for 3 days at 28.5 °C before being subjected to intracellular calcium imaging. Cells grown in L-15 medium were transferred to HEPES-buffered Krebs medium (135 mM NaCl, 5 mM HEPES, 5.4 mM KCl, 1 mM MgCl_2_, 1.8 mM CaCl_2_, 10 mM glucose) and then loaded with fura2-AM (5 µM) for 1 h at 28 °C. The cells were then washed with HEPES-buffered Krebs medium 2-3 times at room temperature. Fluorescence emission was monitored with excitation at 340 and 380 nm using an IX83 (Olympus, Japan), and Ca^2+^ measurements are shown as 340/380-nm ratios obtained from groups of 13-15 cells. Resting Ca^2+^ levels in the three groups were recorded, and then KCl solution was used to stimulate cells to measure the changes in [Ca^2+^]_i_.

**Proteomic and Phosphoproteomic Analyses**

Total proteins were extracted from frozen heart tissue the *tnni3k^e4/+^* heterozygous mutant and WT sibling controls at 6 months. A total of six samples (three biological replicates for each genotype) were sequenced using tandem mass tag (TMT) quantitative proteomics and phosphoproteomics. Peptide preparation and modified protein abundance profiling by Q Exactive^TM^ Plus mass spectrometry（Thermo Scientific^TM^）were performed based on previously described methods^[5]^. Modified protein ratios between groups were calculated by taking the ratios of the total TMT intensities from the corresponding TMT reporter. After classification, 18 components of proteomics were processed, and 6 components of phosphoproteomics were processed. Raw files were processed using Maxquant (Version 1.5.2.8) and searched against the zebrafish protein database (Danio_rerio_7955_PR_20191112) for our analysis, and the parameters were wet to Carbamidomethyl (C) for both proteomic and phosphorylomic fixed modification, while the proteomic modification was Acethyl (Protein-N-term), Deamidation (NQ) and Oxidation (M). Phosphorylation was modified by Acethyl (Protein-N-term), Deamidation (NQ) and Oxidation (M) and phosphorylation (STY). Both the peptide and protein false discovery rate (FDR) cutoff values are set at 0.01. Protein ratios were then log_2_ converted. Proteins significantly changed in the mutant (*tnni3k^e4/+^*) relative to the WT sibling control were determined using *t* tests (two tailed, unpaired). Proteins with a fold change greater than 1.5 and a *P* value less than 0.05 were considered significantly changed. The protein lists of interest were annotated by InterProScan (<http://www.ebi.ac.uk/interpro/>). Putative biological processes/functions and pathways were mapped and generated based on a manually curated knowledge database of molecular interactions extracted from the public literature. The enriched biological processes/functions and KEGG pathways were analyzed using both direct and indirect relationships. GO term annotation/enrichment was carried out using the InterProScn/Perl module:

(<http://www.ebi.ac.uk/interpro/>,<https://metacpan.org/pod/Text::NSP::Measures::2D::Fisher>). KEGG annotation/enrichment was carried out using the InterProScn/Perl module (<http://www.ebi.ac.uk/interpro/>, <https://metacpan.org/pod/Text::NSP::Measures::2D::Fisher>). The phosphorylation-modified motifs were calculated and analyzed using MoMo (<http://meme-suite.org/tools/momo>). A *P* value less than 0.05 was considered evidence of significant enrichment. All the mass spectrometry proteomics raw data have been deposited to the ProteomeXchange Consortium ([https://proteomecentral.proteomexchange.org](http://proteomecentral.proteomexchange.org/)) via the iProX partner repository with the dataset identifier [PXD048620](http://proteomecentral.proteomexchange.org/cgi/GetDataset?ID=PXD048620).

**Western Blotting**

Total, cytoplasmic or nuclear proteins were homogenized and extracted from freshly harvested fish hearts using RIPA solution (Beyotime Biotechnology, Shanghai, China) and an NE-PER^TM^ Kit (Thermo Fisher, 78833) followed by standard Western blot analysis. Briefly, the extracted proteins were separated in 10% polyacrylamide gels and transferred onto nitrocellulose membranes, and the membranes were incubated with primary and subsequently secondary antibodies. The following primary antibodies were used: anti-actin (1:6000, Affinity Biosciences, AF7018), anti-MYPT1 (1:800, Cell Signaling Technology, 2634), anti-YAP1 (1:3000, Proteintech, 13584-1-AP), anti-MLC2 (1:20000, GeneTex, GTX128346), anti-H3 (1:3000, Abcam, 17168-1-AP), anti-Calcineurin (1:800, Abcam, ab210093), anti-NFATC1 (1:800, Fitzgerald, 10R-1130), anti-phospho-YAP1 (1:800, Cell Signaling Technology, 75784), anti-phospho-MYPT1 (1:800, Cell Signaling Technology, 3048), and anti-phospho-MLC2 (1:800, Cell Signaling Technology, 3675). The secondary antibody was purchased from Affinity Biosciences (S0001, S0002). Quantification of relative protein expression was performed using ImageJ software.

**Drug Treatment**

Six-month-old adult zebrafish (*tnni3k^e4/+^* and WT siblings) were incubated with CsA (0.15 µM) or DMSO control twice a day (3 h in the morning and 3 h in the afternoon) for 9 days. On the third day following CsA treatment, cytoplasmic and nuclear proteins were extracted for Western blot analysis.

**Statistical analysis**

Unpaired two-tailed Student’s t tests were used to compare two groups. One-way analysis of variance (ANOVA) was used to evaluate differences among multiple groups. For all dot plots, each value represents the mean ± standard error (SE). The sample size (n) indicates the number of animals otherwise specified. *P* values less than 0.05 were considered to indicate statistical significance. All statistical analyses were carried out using GraphPad Prism 7.0 software.

**References**

1. Thisse C, Thisse B. High-resolution in situ hybridization to whole-mount zebrafish embryos. Nature protocols. 2008; 3: 59-69.

2. Ding Y, Lang D, Yan J, Bu H, Li H, Jiao K, et al. A phenotype-based forward genetic screen identifies Dnajb6 as a sick sinus syndrome gene. Elife. 2022; 11.

3. Wang LW, Huttner IG, Santiago CF, Kesteven SH, Yu ZY, Feneley MP, et al. Standardized echocardiographic assessment of cardiac function in normal adult zebrafish and heart disease models. Dis Model Mech. 2017; 10: 63-76.

4. Sander V, Suñe G, Jopling C, Morera C, Izpisua Belmonte JC. Isolation and in vitro culture of primary cardiomyocytes from adult zebrafish hearts. Nature protocols. 2013; 8: 800-9.

5. Song G, McReynolds MR, Walley JW. Sample Preparation Protocols for Protein Abundance, Acetylome, and Phosphoproteome Profiling of Plant Tissues. Methods in molecular biology (Clifton, NJ). 2017; 1610: 123-33.
