# Supplementary material for: Tnni3k regulates cardiomyopathy and cardiac conduction disease through Nfatc1 signaling
Source: Genes Dis. 2024 Nov 13;12(3):101464. doi: 10.1016/j.gendis.2024.101464 (PMC11804685; doi:10.1016/j.gendis.2024.101464)

Supplementary Figure S1. The zebrafish Tnni3k protein shares high homology with human TNNI3K

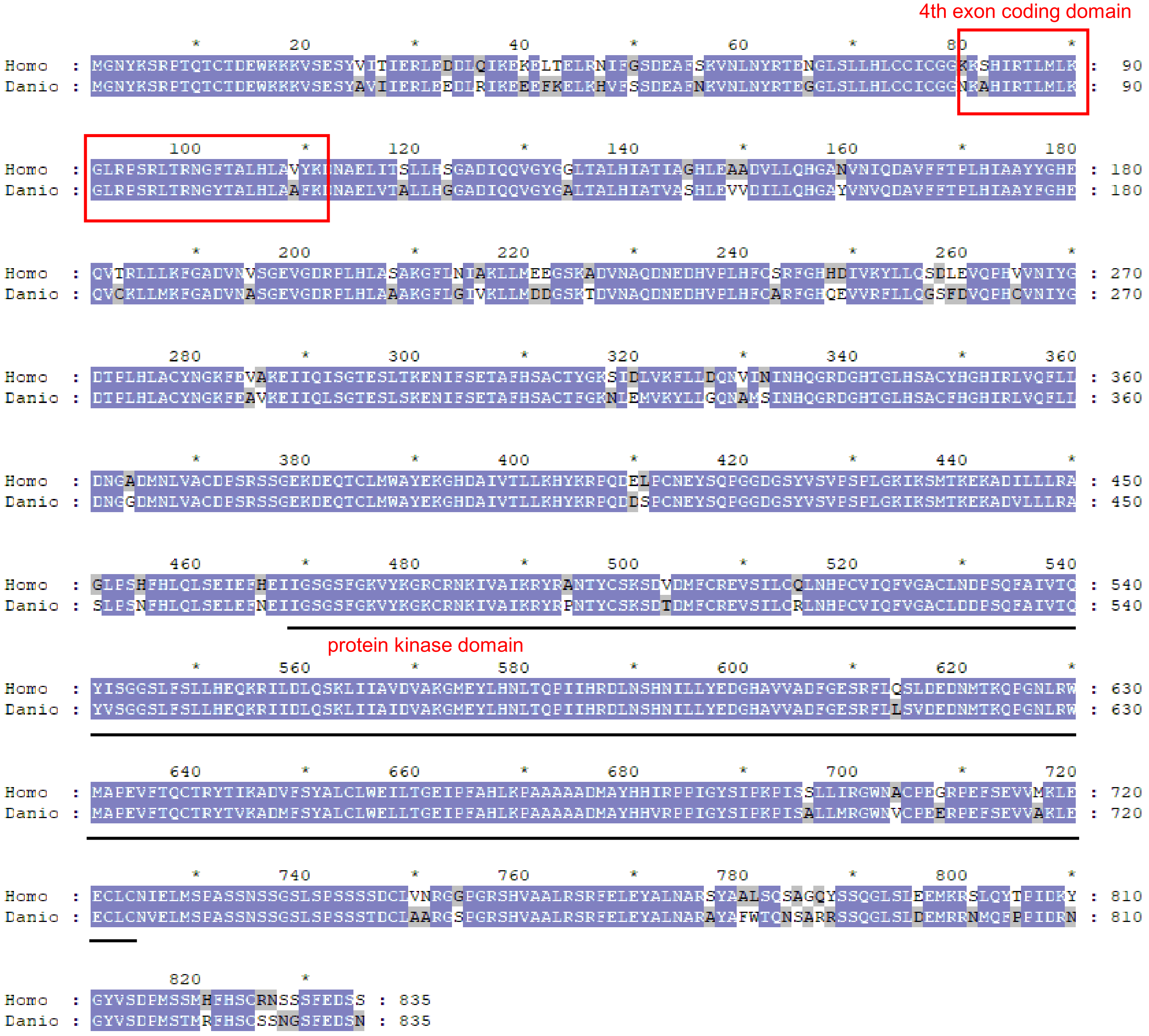

Supplementary Figure S2. Generation of the *tnni3k* splicing mutant which exhibited enlarged ventricular chamber size. **(A)** Schematic illustration of the single guide RNA (sgRNA) sequence design targeting the 4th exon of the zebrafish *tnni3k* gene and positions of primers for genotyping PCR and transcript analysis. The sgRNA sequence is shown in green, and the PAM (protospacer adjacent motif) site is underlined in red. The primer pairs of F and R were used for genotyping. The primer pairs of F1 and R1 and F2 and R2 were used for transcript analysis. (**B)** Representative DNA gel images of semiquantitative RT–PCR to analyze the *tnni3k* transcript between WT and mutant alleles. **(C)** At the transcription levels, chromographs illustrating the sequences of the WT *tnni3k* gene and the mutant alleles with a 6-base pair (bp) nucleotide deletion in the splicing site adjacent between the 4th exon and 5th intron which leads to a premature stop (red box). **(D-E)** Representative images of dissected hearts (D) and quantification analysis of ventricular surface area (VSA) (E), and VSA/body wight (BW) ratio (F) from the indicated fish at 6 months. Significantly enlarged ventricles were noted in the *tnni3k* mutants. Scale bars, 500 μm. N=3, one-way ANOVA.

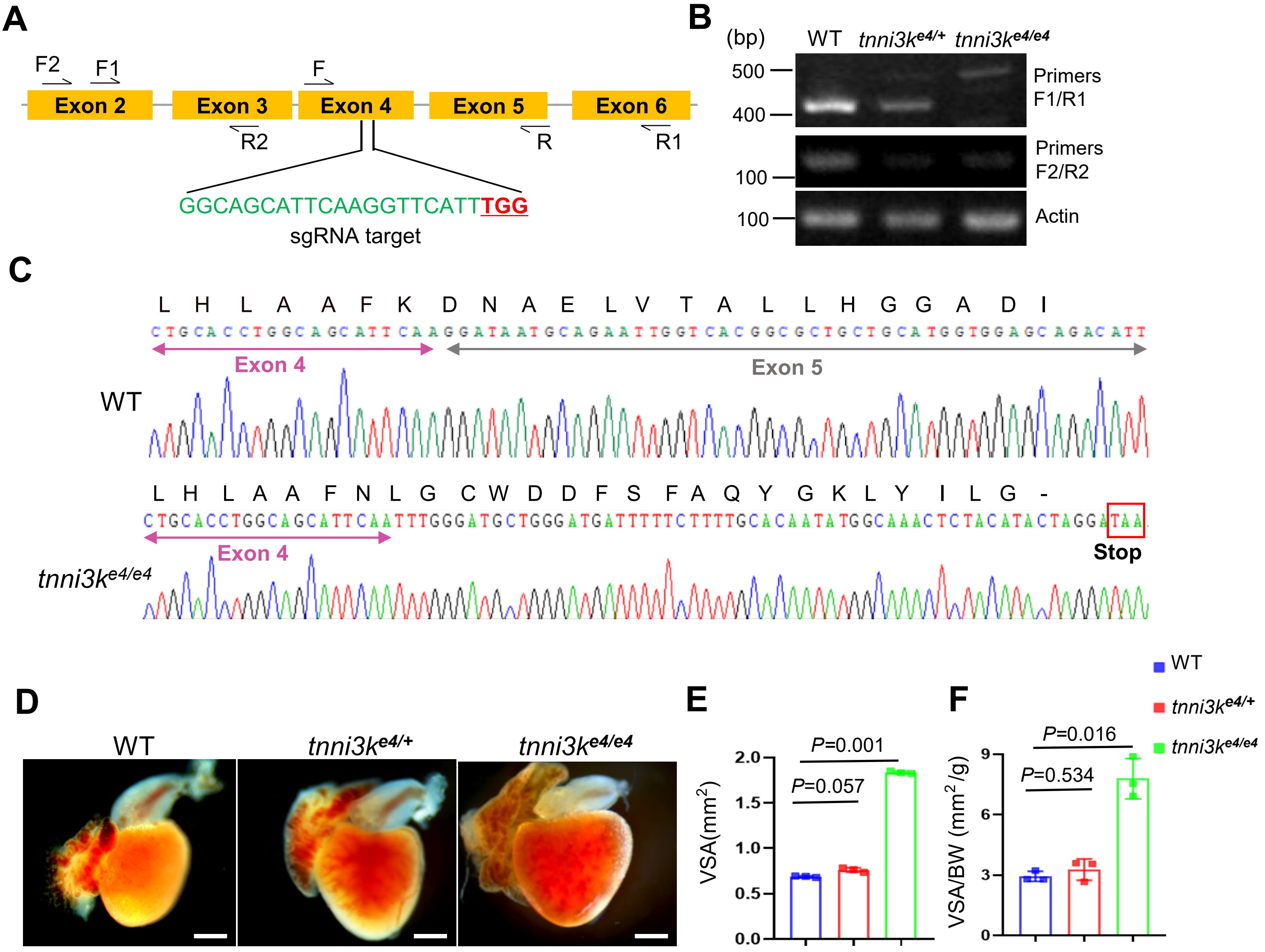

Supplementary Figure S3. Proteomic and phosphoproteomic analysis identified differentially expressed phosphorylated proteins in the *tnni3k*$^{e4/+}$ mutant hearts. **(A-B)** Principal component analysis (PCA) (A) and volcano plot of changes in protein levels (B) in the *tnni3k*$^{e4/+}$ mutant hearts versus in the WT controls. **(C-D)** PCA (C) and volcano plot of changes in protein phosphorylation levels (D) in the *tnni3k*$^{e4/+}$ mutant hearts versus in the WT controls. **(E)** Western blotting quantification analysis of the expression levels of indicated proteins in the *tnni3k*$^{e4/+}$ mutant hearts compared to WT control hearts at 6 months. N=3-4, one-way ANOVA.

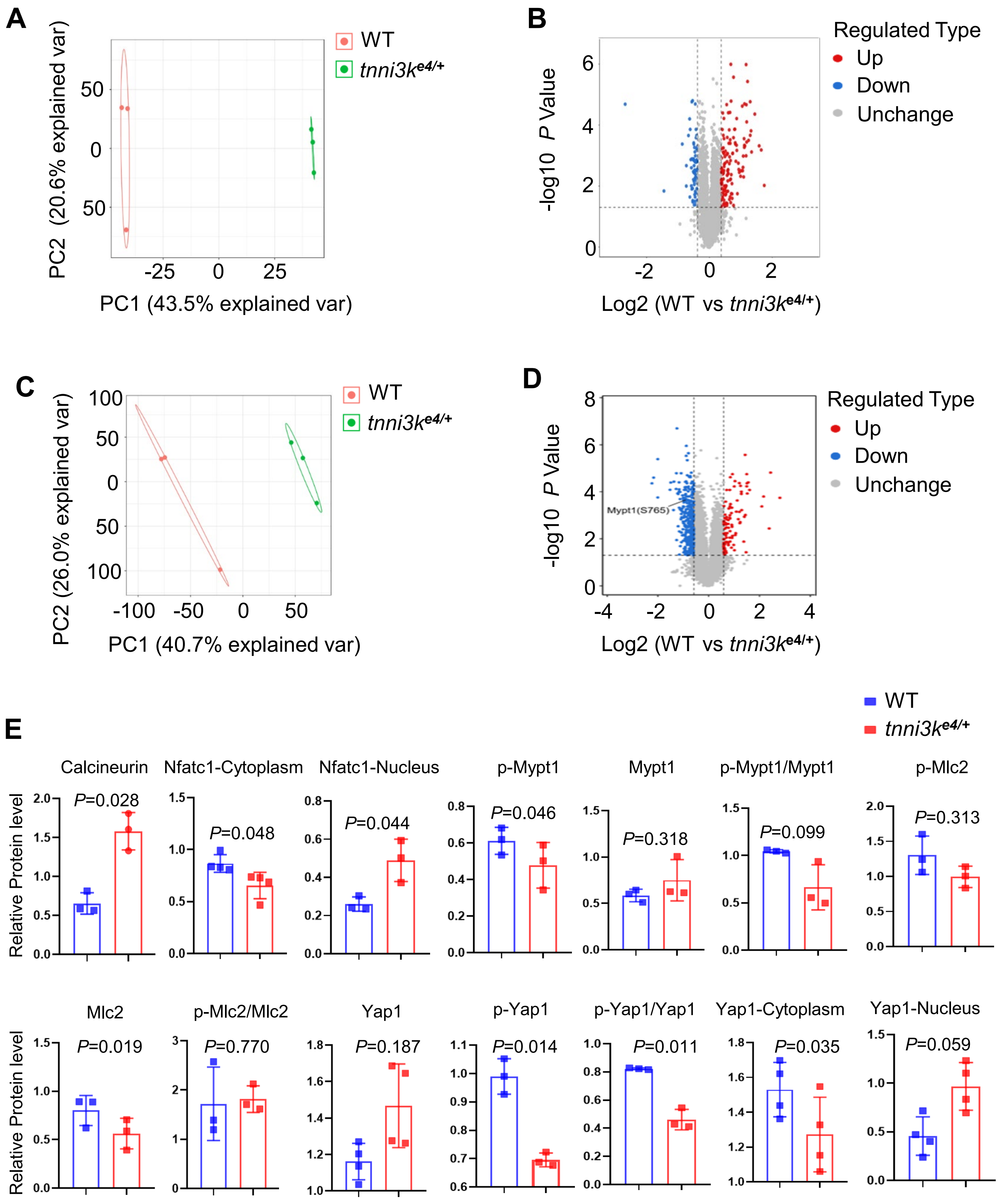

Supplementary Figure S4. CsA treatment inhibited the Nfatc1 protein nuclear translocation. **(A-B)** Representative Western blotting (A) and quantification analysis (B) of the expression levels of nuclear versus cytoplasmic Nfatc1 proteins in the *tnni3k*$^{e4/+}$ mutant hearts compared to WT control hearts with cyclosporine A (CsA) or DMSO treatment at 6 months . N=3 biological replicates, one-way ANOVA.

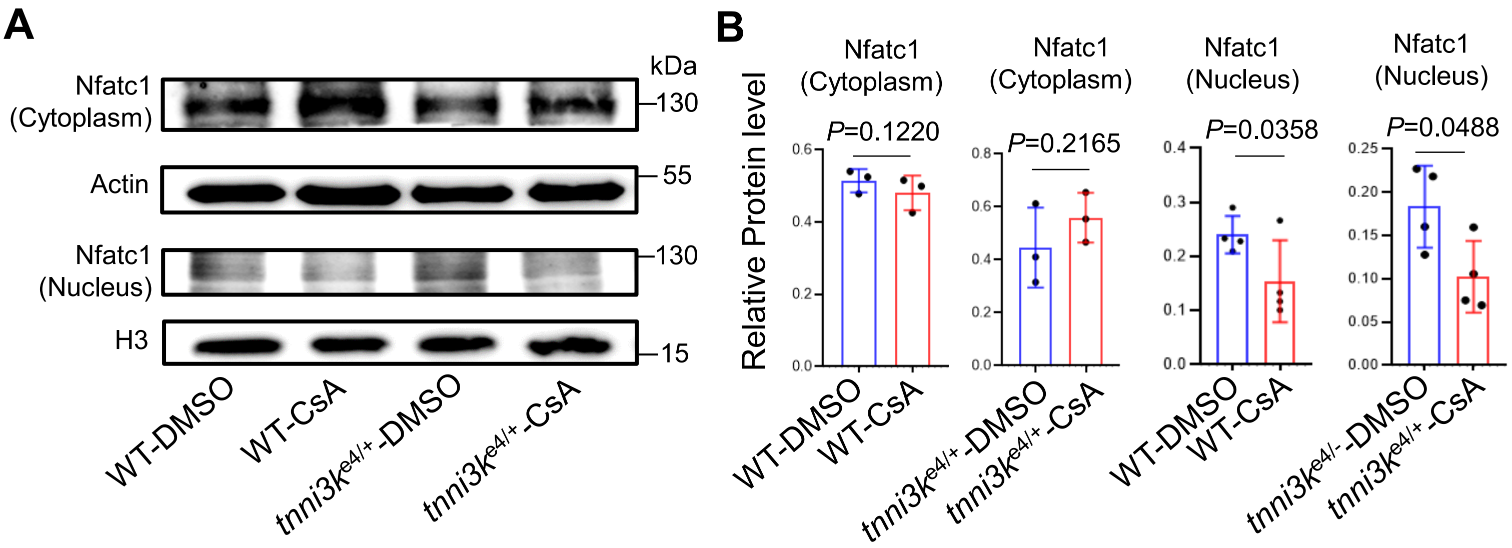

Supplement: Multimedia component 2 [file mmc2.pdf]
